# Supplementary material for: Comparison of Three Dietary Assessment Methods to Estimate Meat Intake as Part of a Meat Reduction Intervention among Adults in the UK
Source: Nutrients. 2022 Jan 18;14(3):411. doi: 10.3390/nu14030411 (PMC8839883; doi:10.3390/nu14030411)
Supplement: Supplementary file 1 [file nutrients-14-00411-s001.zip › nutrients-1531319-supplementary.pdf]

Supplementary Figure S1. Bland-Altman plots for red and processed meat, and unprocessed white meat intake (g/day) showing the comparability of three methods to estimate meat intake.

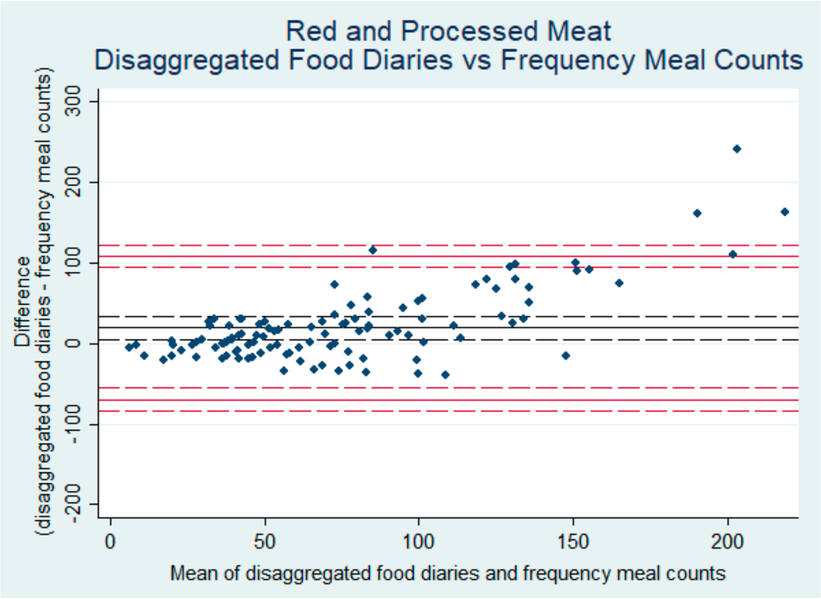

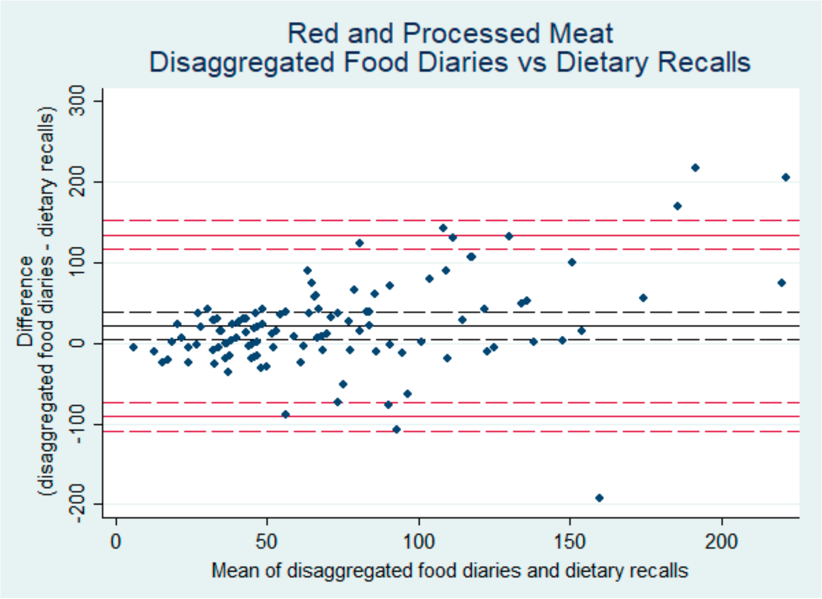

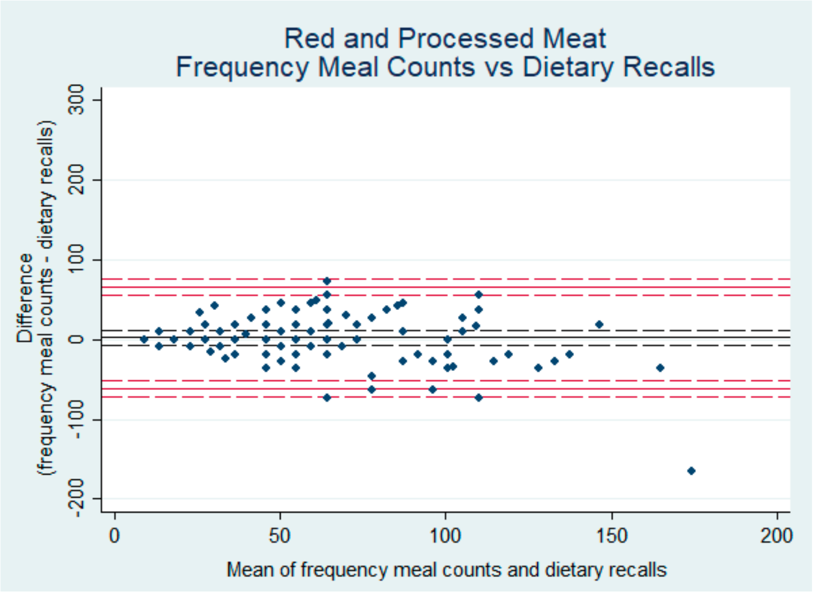

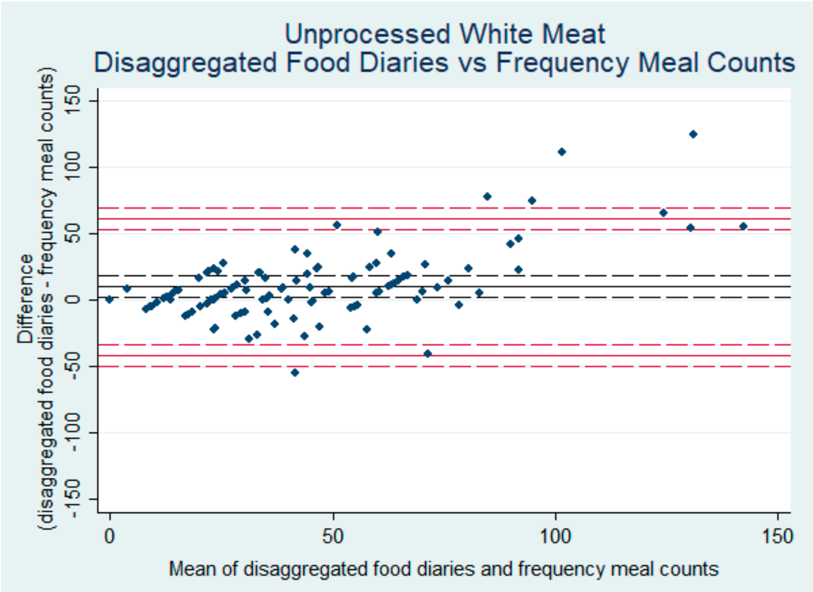

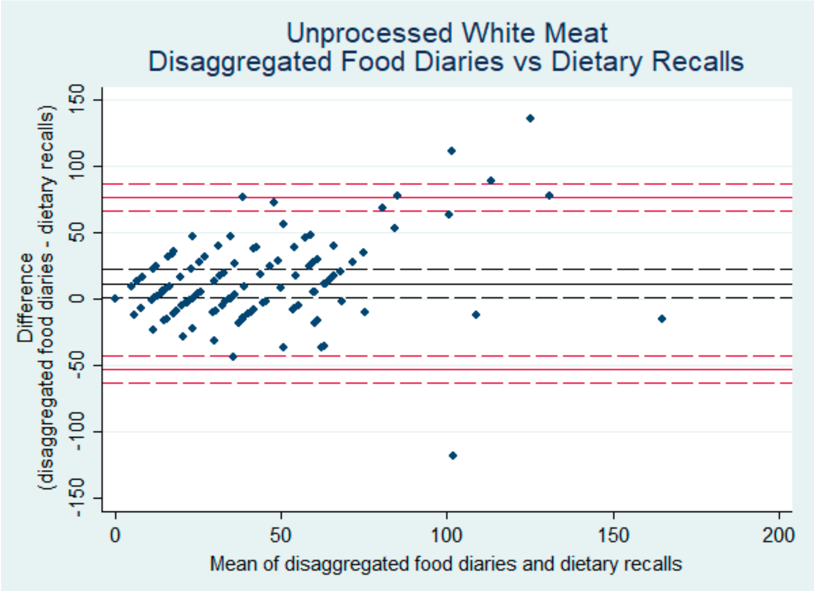

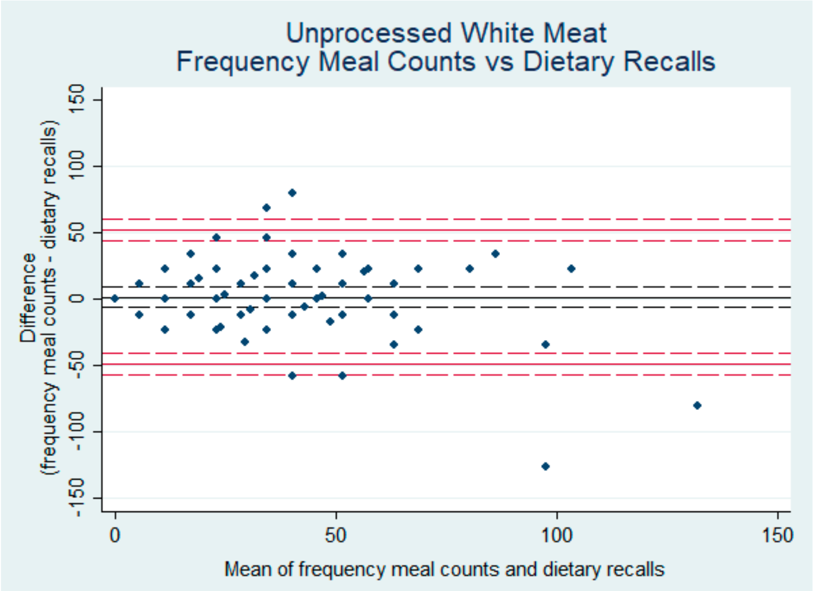

Disaggregated food diaries: disaggregating the quantity of meat from meat-containing composite products recorded in 7-day food diaries; Frequency meal counts: counting the frequency of meals containing meat recorded in 7-day food diaries*standard portion size; Dietary recalls: asking participants to retrospectively recall how often they consumed meat in the last 7 days through a questionnaire*standard portion size. Solid red lines are the limits of agreement with 95% confidence intervals (red dashed lines). The solid black line is the mean difference (bias) together with the 95% confidence intervals (black dashed lines).

Supplementary Table S1. Comparison of standard portion sizes with actual portion sizes obtained from food diaries (g)

	**Standard**	**Food diaries**
Total meat	69.3	89.5
Red and processed meat	64.1	83.8
Unprocessed white meat	80.3	101.5

Standard portion sizes underlying the two frequency dietary measures were obtained from a specific meat frequency questionnaire which utilised information from the UK Food Standards Agency's food portion size book and meat disaggregation data from the food composition database of the UK's National Diet and Nutrition Survey, Year 10[1]. Food diary portion sizes were calculated by dividing the daily average disaggregated consumption by the daily average frequency meal count from the 7-day food diaries.

1. Stewart, C.; Frie, K.; Piernas, C.; Jebb, S.A. Development and Reliability of the Oxford Meat Frequency Questionnaire. *Nutrients*. **2021**, *13,* 922.
